# Impact of social support on the resilience of youth: mediating effects of coping styles

**DOI:** 10.3389/fpubh.2024.1331813

**Published:** 2024-03-20

**Authors:** Fei Cao, Juan Li, Wei Xin, Nan Cai

**Affiliations:** ^1^Department of Sociology, School of Law, Jiangnan University, Wuxi, China; ^2^Department of Medical Psychology, The Sixth Medical Center of PLA General Hospital, Beijing, China; ^3^Student Mental Health Education and Counseling Center, Xi'an International Studies University, Xi'an, China

**Keywords:** social support, resilience, coping styles, the youth, mental health

## Abstract

**Background:**

Chinese youth are at high risk for depression with a significantly higher detection rate of depression risk than other age groups, which brings about a huge challenge to the mental health work of universities. Developing supportive resources that promote resilience against adverse environmental influences in high-risk groups is quite more urgent than medical treatment for firm diagnoses of mental issues that have developed into depression in the current background.

**Methods:**

A total of 665 university students in China completed self-reported questionnaires measuring psychological resilience, social support, and coping styles. The structural equation model testing on the goodness of fit of the theoretical framework was first performed. Descriptive statistics and Pearson’s correlation analysis among social support, resilience, and coping styles were then conducted. At last, we tested the mediating role of coping styles.

**Results:**

Social support has a significant positive effect on the psychological resilience of the youth. Mixed coping and immature coping styles have significant negative impacts on both social support and resilience, while mature coping styles have a significant positive effect on social support and resilience. Mature and immature coping styles mediate the association between social support and resilience in youth.

**Conclusion:**

Based on stress theory, this study explores mechanisms that facilitate the development of resilience in young people with regard to social support and coping styles. The current research depicts an interventional perspective of building a social support network that guides the youth to adopt mature coping styles to enhance their resilience and facilitate their mental health.

## 1 Introduction

The mental health issues of students in higher education are tending to be a new challenge in public health (1). In 2022–2023, over 40% of students in higher education had clinically significant symptoms of depression in America (2), the trend of which was declared as a mental health crisis by the United States Surgeon General (3). In China, this trouble is equally prominent. According to the survey results of the Report on National Mental Health Development in China (2021–2022), the risk of depression and anxiety among young people is higher than that of other age groups. The detection rate of depression risk in the age group of 18–24 years old was as high as 24.1%, with nearly 50% of them being students (4). Paradoxically, dramatic social changes in recent years have ushered in a great shortage of mental health services (5) while the stigmatization of mental illness discourages people from seeking help (6–8). In such a background, it is more crucial to provide positive support to enhance the youth’s mental resources that help prevent psychological symptoms from developing into mental disorders compared to conventional treatments.

The current study was conducted with a focus on mental health protective factors. We aimed to shed light on the impact of social support on the resilience of youth, taking coping styles as mediating variables to examine the mechanisms of action that individuals apply to cope with stress. The further goal of this study is to offer valuable insights into the evidence and support for the development of psychological service systems for high-risk groups.

### 1.1 The effect of social support on resilience

Social support and resilience are considered mental health protective factors because they facilitate positive adaptations to adversity and sustain post-trauma growth (9–11). As a positive mental feature, resilience helps individuals counter negative influences of stressors and allows them to cope with adversities or stressful events in a better way, experience fewer negative emotions, and gain a higher level of subjective wellbeing (12–14). Extensive research has confirmed the crucial role resilience has played in promoting mental health and preventing mental illnesses. In the mental health field, resilience was regarded as the protective factor against psychological issues such as loneliness (15) and pressure (16). In studies of students, resilience could effectively predict wellbeing (16, 17) and adjustment to university life (18, 19), leading more and more scholars to advocate intervention strategies that focus on increasing resilience to decrease the risk of mental illnesses (15, 17). According to the conservation of resources theory proposed by Hobfoll, resilience is defined as having abundant mental and social resources in a stressful condition (20), which means resilience helps individuals deploy all their resources to overcome challenging situations. Therefore, high-resilient individuals are better at leveraging various resources to cope with impossibilities and setbacks and could adapt better to stressful conditions (21). Social support, as an essential external resource, plays a fundamental role for individuals to handle stressful environments and incidents in their lives. A multitude of empirical studies have found a positive correlation between resilience and social support. In studies of students, those who perceived more social support reported higher resilience (17), and demonstrated better adaptation to new environments (22). Social support can alleviate the harmful impacts that stressful events have on individuals, and promote resilience to exert a positive influence on psychological wellbeing (23). Thus, we proposed the following hypothesis:

*H*1: Social support positively affects psychological resilience in youth.

### 1.2 The mediating effects of coping styles

Currently, an increasing number of scholars believe that mental health is the process by which resilience comes into play, with the outcomes determined by the interaction between the features of individuals and their coping styles (24). In coping with stress, individuals tend to adopt different cognitive and behavioral efforts to manage potential threats and effectively reduce the impact that stress and its adverse consequences have on personal resources (25). Several studies have revealed that adopting suitable coping styles demonstrates a noteworthy positive correlation with a higher level of resilience (19, 23), and accordingly fosters positive outcomes of mental health and psychological wellbeing (26–28). In the existing literature, social support can enhance the resilience of medical staff through positive coping strategies in public health emergencies (29). Similar findings have also been spotted in other clinical research. In Haase’s study, social support could significantly affect the resilience of adolescents and young adults with cancer through courageous coping (30). This implies that coping styles could probably play a mediating role in the correlation between social support and resilience. Thus, we proposed the following hypothesis:

*H*2: Coping styles mediate the correlation between social support and resilience.

**Figure 1:**
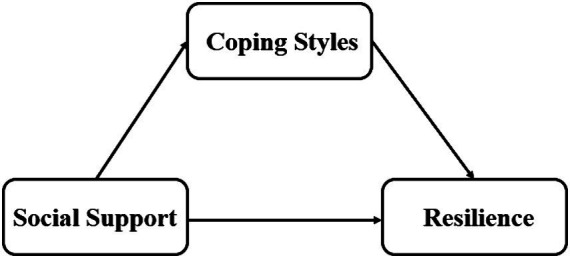
Theoretical framework.

Figure 1 depicts the theoretical framework for this study.

## 2 Methods

### 2.1 Participants and recruitment

This study adopts convenience sampling to conduct a questionnaire-based survey with participants recruited from a university in southern China. The survey was anonymous, and all the participants were made aware of and consented to the objectives of the study before they took the questionnaire. A total of 717 questionnaires were collected with 665 being effective, comprising a response rate of 92.7%.

### 2.2 Measures

#### 2.2.1 Demographics

We used a demographic questionnaire to collect demographic data, which included five parameters: (a) gender, (b) age, (c) major, and (d) school year.

#### 2.2.2 Measurement of resilience

The Connor-Davidson Resilience Scale (CD-RISC) (31) was adopted to measure resilience. The scale comprises 25 items, each rated on a 5-point Likert scale, with 0 indicating never, 1 seldom, 2 sometimes, 3 often, and 4 almost always. Total scores ranged from 0 to 100, with higher scores indicating greater resilience. This scale has been tested with solid reliability and validity and has been widely used in both clinical practice and psychological research (29, 32, 33). The internal consistency reliability of this study stood at 0.932.

#### 2.2.3 Measurement of social support

The Social Support Rating Scale developed by Shuiyuan (34), which presented sound psychometric properties in the Chinese population (35), was adopted to measure social support. It consists of 10 items with three dimensions: subjective social support, objective social support, and utilization of social support. Subjective social support refers to personal emotional experience and satisfaction about the respect, support, and understanding that an individual gains. It consists of four items [e.g., How many close friends do you have, and from whom you can gain support and help? (1) None, (2) 1–2, (3) 3–5, and (4) 6 and above]. Objective social support refers to specific or valuable assistance, including direct material support, community relationships, and group participation. It comprises three items (e.g., Over the past year, you have been (1) staying away from your family and living alone; (2) traveling too much and spending most of your time with strangers; (3) living with friends from school, work, or other places; and (4) living with your family). Utilization of social support refers to an individual’s positive usage of various forms of social support, including ways of communication and seeking help as well as participating in events. It consists of three items (e.g., What do you do when you are bothered about something? (1) You never tell anybody about your troubles; (2) You only share your troubles with one or two close friends; (3) You tell your friends if they ask; and (4) You openly communicate your troubles to gain support and understanding). The total scores on the scale ranged from 12 to 66, with higher scores indicating a higher level of social support. The internal consistency reliability for the three dimensions mentioned above stood at 0.646, 0.597, and 0.575, respectively.

#### 2.2.4 Measurement of coping styles

The Coping Styles Questionnaire (CSQ) (36), which was developed according to Folkman and Bond’s coping and defense questionnaires (37, 38), was adopted to examine the coping styles of the youth. This questionnaire has been primarily used to assess coping in the context of Chinese language features with solid reliability and validity (26, 39). The questionnaire consists of subscales (problem-solving, self-blame, help-seeking, fantasizing, avoidance, and rationalization) and 62 items, each rated 0 (agree) or 1 (disagree). Among the six subscales, problem-solving and help-seeking were considered mature coping styles; avoidance, fantasizing, and self-blame were regarded as immature coping styles; and rationalization was considered as the mixed coping style. In this study, the internal consistency reliability of the six sub-scales ranged from 0.776 to 0.899.

## 3 Data analysis

Analysis of the data was conducted utilizing IBM SPSS 23.0 and AMOS 24.0. The structural equation model testing on the goodness of fit of the theoretical framework was first conducted through AMOS 24.0. Then we performed the Harmon single-factor test to detect the common method bias (40). Descriptive statistics and Pearson’s correlation analysis among social support, resilience, and coping styles was conducted through IBM SPSS 23.0. The mediating role of coping style was tested through the SPSS macro program PROCESS 3.5 developed by Hayes (Model 4) (41).

## 4 Results

### 4.1 Testing of the goodness of fit of the theoretical framework

The hypothesized relationships of the study’s framework were tested in the structural model through AMOS 24.0, the results of which have been depicted in Table 1. The results indicate a good fit of the theoretical model with χ^2^/df = 2.507, SRMR = 0.050, RMSEA = 0.048, GFI = 0.901, AGFI = 0.886, IFI = 0.922, CFI = 0.921, TLI = 0.915. Therefore, the hypothesized framework was a good fit for the empirical data (42).

**Table 1:** The main indicators of the model fit test (*N* = 665).

Fit indices	χ^2^/df	SRMR	RMSEA	GFI	AGFI	IFI	CFI	TLI
Reference values	<3	<0.08	<0.08	>0.90	>0.90	>0.90	>0.90	>0.90
Goodness-of-fit	2.507	0.050	0.048	0.901	0.886	0.922	0.921	0.915

### 4.2 Control and testing for common method bias

As all questionnaires used in this study were self-rating scales, there may have been common method bias. To detect this, we adopted the Harmon single-factor test. The results showed a total of 21 factors with eigenvalues greater than one and 19.81% of the variances being explained by the first factor, which were less than the critical standard of 40%. This indicated that there was no significant common method bias in this study (40).

### 4.3 Descriptive statistics and correlation analysis

Table 2 depicts the descriptive statistics. More than half of the participants were female (64.66% of the total), with an average age of 20.36 ± 1.81 years old. In terms of major, most of the participants majored in social sciences (88.57%).

**Table 2:** Descriptive statistics of the participants (*N* = 665).

Variable	*N*	Percent (%)/Mean ± SD
Gender		
Male	235	35.34%
Female	430	64.66%
Age		20.36 ± 1.81
Major		
Social work	243	36.54%
Accounting	178	26.77%
Finance	168	25.26%
Other majors	76	11.43%
School year		
Freshman	267	40.15%
Sophomore	162	24.36%
Junior	142	21.35%
Senior	94	14.14%

Table 3 displays the results of the correlation analysis. The analysis shows that social support had a significant positive correlation with resilience (*p* < 0.01), indicating that social support was an essential fortifying factor for resilience and might thus promote resilience against adversities and stressful circumstances. Hypothesis 1 was supported. Mixed and immature coping styles had a significant negative correlation with resilience (*p* < 0.01), while mature coping styles demonstrated significant positive correlations with resilience (*p* < 0.01). Mixed and immature coping styles displayed significant negative correlations with social support (*p* < 0.01), while mature coping styles showed significant positive correlations with social support (*p* < 0.01). The results of correlation analysis indicate that different coping styles have different effects on resilience. Compared with mixed and immature coping styles, mature coping styles can effectively enhance individuals’ resilience.

**Table 3:** Correlation analysis of major factors (*N* = 665).

Variables	*M*	*SD*	1	2	3	4	5
1. Resilience	77.408	14.586	–				
2. Mixed coping style	4.382	2.439	−0.253**	–			
3. Mature coping styles	17.808	2.585	0.325**	−0.058	–		
4. Immature coping styles	10.651	7.463	−0.331**	0.856**	−0.142**	–	
5. Social support	42.414	6.610	0.435**	−0.258**	0.386**	−0.315**	–

**p < 0.01.

### 4.4 Test for the mediating effects of coping styles

To test the mediating effect that coping styles play in the relationship between social support and resilience, we conducted a bootstrapping analysis with 5,000 resamples (41, 43). See Table 4 for detailed results. The analysis showed that social support had a significant positive impact on mature coping styles (*β* = 0.386, *p* < 0.01) and a negative effect on the mixed coping style (*β* = −0.258, *p* < 0.01) and immature coping styles (*β* = −0.315, *p* < 0.01). Meanwhile, the impact that social support had on resilience is also statistically significant (*β* = 0.301, *p* < 0.01). The mediating role of coping style was tested through the SPSS macro program PROCESS 3.5, the results of which indicated a significant positive impact mature coping styles having on resilience (*β* = 0.175, *p* < 0.01) while a negative effect with the immature coping style (*β* = −0.259, *p* < 0.01). Accordingly, the relationship between social support and resilience was mediated by coping styles (mature and immature coping styles), indicating that coping styles were the mechanism by which the effect of social support had on resilience. These results supported Hypothesis 2.

**Table 4:** Bootstrapping analysis of the mediating effect of coping styles.

Process	Variable	Model 4					
		*R* ^2^	*F*	*β*	*SE*	*t*	95% CI
1. Mediator variable model (CS)							
MICS	SS	0.066	47.119	−0.258	0.014	−6.864^**^	[−0.122, −0.068]
MCS		0.149	116.020	0.386	0.014	10.771^**^	[0.123, 0.178]
IMCS		0.099	73.211	−0.315	0.042	−8.556^**^	[−0.438, −0.274]
2. Dependent variable model (RIS)	SS			0.301	0.084	7.929^**^	[0.499, 0.827]
	MICS			0.056	0.390	0.857	[−0.432, 1.101]
	MCS			0.175	0.207	4.790^**^	[0.584,1.395]
	IMCS			−0.259	0.130	−3.897^**^	[−0.761, −0.251]
*R*^2^ = 0.259, *F* = 57.718							

CS, coping style; RIS, resilience; SS, social support; MICS, mixed coping style; MCS, mature coping styles; IMCS, immature coping styles; CI, confidence interval. **p < 0.01.

## 5 Discussion and conclusion

Youth populations are facing unprecedented mental health challenges (1–4). Compared to conventional medical treatments, it is more crucial to provide positive support to enhance the youth’s mental resources that help prevent psychological symptoms from developing into mental disorders. Resilience, social support, and coping styles have important implications for mental health. The current study explored the interrelationships among resilience and internal (coping styles) and external (social support) resources, trying to find an interventional mechanism of how the protective factors operate. The research findings showed that social support and coping styles had significant correlations with resilience, and the hypothesized framework manifested a good fit. Resilience of the youth can be positively predicted by social support and mature coping styles, while negatively predicted by mixed and immature coping styles. Our findings are consistent with studies that have verified the significant effects these factors exert in buffering adverse outcomes in different populations (13, 14, 23, 29), which confirms the protective functions of social support and mature coping styles to psychological wellbeing.

Our study has also developed a theoretical framework in which coping styles were considered as the mediating mechanisms that act on resilience through social support. We found that mature coping styles (problem-solving and help-seeking) were significantly and positively related to social support and resilience while immature coping styles (avoidance, fantasizing, and self-blame) showed a significant effect otherwise. Consistent with previous research, mature coping styles often promote higher resilience and good adaptation in stressful situations (15, 17, 19). However, the mediating effect of coping styles showed inconsistency in studies of varied populations. In our study, the mediating effects of both mature and immature coping styles were significant, with an insignificant mediating effect of the mixed coping style. In adolescents with hemophilia, the mediating effect of positive coping barely influenced the relationship between social support and resilience (28). In adult population, however, positive coping played a significant role in mediating social support and resilience-related features (44, 45). The inconsistency may result from the possibility that the coping styles of different populations are influenced by personal traits and diverse cultural environments (24, 39, 46). In studies of military groups, negative coping styles were commonly used because of the advocated culture that urged military personnel to operate proficiently in stressful environments (47).

The current research focused on the protective factors of mental health. We carried out an initial exploration into the relationships among social support, coping styles, and resilience in the youth, trying to find an interventional mechanism of how the protective factors operate. Our findings indicated that mental health interventions, which aim to help the youth develop resilience, should encourage individuals to adopt mature coping styles, such as seeking help and avoiding the use of immature coping styles, such as self-blame and avoidance, in stressful situations. In this way, the youth can promote psychological wellbeing in a more effective way.

However, this study has limitations. Firstly, some of our findings are inconsistent with previous research. Several studies have revealed that demographics such as gender and major would influence individuals’ coping mechanisms and resilience (17, 19). Therefore, identifying key demographic variables that affect the protective factors of mental health may be of great benefit for accurate intervention in preventing mental illness. Secondly, this study found that the mixed coping styles (rationalization) had a significant positive correlation with immature coping styles, which is inconsistent with findings that the mixed coping styles demonstrated a significant negative correlation with mature coping styles (48). It probably indicated that when coping with a stressful situation, as a mixed coping style, rationalization may operate through different mechanisms and thus exert separated influences. It is necessary to further discuss if there are prioritized coping strategies in different groups in future research. Lastly, considering the limitations of cross-sectional studies, future studies should leverage longitudinal research and experiments to confirm the interrelationships of the protective factors and provide more convincing evidence and more reliable guidance for the implementation of psychological services in practice.

## Data availability statement

The datasets presented in this article are not readily available because of participant privacy and ethical requirements. Requests to access the datasets should be directed to caofei@jiangnan.edu.cn.

## Ethics statement

The studies involving humans were approved by the Ethics Committee of Jiangnan University. The studies were conducted in accordance with the local legislation and institutional requirements. The participants provided their written informed consent to participate in this study.

## Author contributions

FC: Writing – original draft. JL: Supervision, Writing – review & editing. WX: Formal Analysis, Writing – original draft. NC: Investigation, Writing – original draft.
